# The Equity Impact of Universal Home Visits to Pregnant Women and Their Spouses in Bauchi State, Nigeria: Secondary Analysis From a Cluster Randomised Controlled Trial

**DOI:** 10.1177/2752535X241249893

**Published:** 2024-04-27

**Authors:** Anne Cockcroft, Loubna Belaid, Khalid Omer, Umaira Ansari, Amar Aziz, Yagana Gidado, Hadiza Mudi, Rilwanu Mohammed, Rakiya Sale, Neil Andersson

**Affiliations:** 1 CIET-PRAM, Department of Family Medicine, 5620 McGill University, Montreal, QC, Canada; 2 Centro de Investigácion de Enfermedades Tropicales, 341132 Universidad Autónoma de Guerrero, Acapulco, Mexico; 3 113642 École Nationale d’Administration Publique, Montreal, Canada; 4 Federation of Muslim Women’s Associations of Nigeria (FOMWAN), Bauchi, Nigeria; 5 Bauchi State Primary Health Care Development Agency, Bauchi, Nigeria; 6 Bauchi State College of Nursing and Midwifery, Bauchi, Nigeria

**Keywords:** maternal health, health equity, home visits, universal health coverage, implementation research

## Abstract

**Background:**

Socio-economically disadvantaged women have poor maternal health outcomes. Maternal health interventions often fail to reach those who need them most and may exacerbate inequalities. In Bauchi State, Nigeria, a recent cluster randomised controlled trial (CRCT) showed an impressive impact on maternal health outcomes of universal home visits to pregnant women and their spouses. The home visitors shared evidence about local risk factors actionable by households themselves and the program included specific efforts to ensure all households in the intervention areas received visits.

**Purpose:**

To examine equity of the intervention implementation and its pro-equity impact.

**Research design and study sample:**

The overall study was a CRCT in a stepped wedge design, examining outcomes among 15,912 pregnant women.

**Analysis:**

We examined coverage of the home visits (three or more visits) and their impact on maternal health outcomes according to equity factors at community, household, and individual levels.

**Results:**

Disadvantaged pregnant women (living in rural communities, from the poorest households, and without education) were as likely as those less disadvantaged to receive three or more visits. Improvements in maternal knowledge of danger signs and spousal communication, and reductions in heavy work, pregnancy complications, and post-natal sepsis were significantly greater among disadvantaged women according to the same equity factors.

**Conclusions:**

The universal home visits had equitable coverage, reaching all pregnant women, including those who do not access facility-based services, and had an important pro-equity impact on maternal health.

## Introduction

The World Health organization describes health inequities as “differences in health status or in the distribution of health resources between different population groups, arising from the social conditions in which people are born, grow, live, work and age”.^1^ The COVID-19 pandemic highlighted and brutally exacerbated long-standing health inequities.^2^ Public health and other interventions, particularly those targeting individual behaviours or only applying to those who can access health facilities, may improve health for some but worsen health inequity.^3^ There are huge disparities in maternal morbidity and mortality between countries, and these have increased over the last 5 years.^4^ Within high-income countries, socio-economically disadvantaged women have worse health outcomes overall (such as disability free life expectancy),^5^ and worse maternal health outcomes.^6,7^ In low- and middle-income countries (LMICs), many of the same structural factors (such as poverty and lack of education) and other inequity factors (such as ethnicity and area of residence) that increase maternal health risks also limit access to facility-based antenatal care and other maternal health services.^8-10^ Maternal health services in LMICs are often also under-resourced and over-stretched and unable to provide adequate care even if women do access them.^11^ A review of reviews cautioned that some strategies to improve quality of maternal and child health care in LMICs might worsen inequities; for example strategies providing information and training online can be effective in urban communities but the materials are not accessible in poor rural communities without internet access.^12^

National and international bodies prioritise health equity.^13,14^ A review of interventions to reduce inequalities in maternal and child health in LMICs concluded those more likely to be effective included outreach services, use of local human resources, and provision of nearby community services.^15^ A review of the effects of community health worker interventions in LMICs found that some but not all studies reported pro-equitable coverage; community health workers were more likely to improve equity in maternal and child health outcomes if they included engagement with community leaders.^16^ Both reviews called for analyses of equity impact of interventions for improving maternal and child health and for more contextual and process information about interventions that improve equity.^15,16^

A stepped-wedge cluster randomized controlled trial of a program of universal home visits to pregnant women and their spouses in Bauchi State, Nigeria, reported improvements in maternal and child health outcomes and in male attitudes and behaviours.^17-20^ The present analysis assessed equity of implementation of home visits and examined whether their impact on maternal health outcomes differed according to three equity factors: rural community, extreme household poverty (insufficient food), and no formal maternal education.

## Methods

### Setting

Maternal mortality in Nigeria is among the highest in the world, with an estimated maternal mortality ratio (MMR) of 1,047deaths per 100,000 live births in 2020, representing 82,000 maternal deaths per year; this implies very little decrease since 2000 (MMR 1148).^4^ Subnational data are sparse but suggest that maternal mortality in the northeastern Bauchi State is even higher than the Nigerian national figure; a study in Bauchi secondary health facilities reported a facilities-based MMR of 4113 in 2010.^21^ Bauchi State has an estimated population of about 4.6 million, mainly Muslim and of Hausa ethnicity. Some 63% of women and 44% of men have no formal education, compared with 35% and 22% respectively at national level, and only 12% of Bauchi women participate in household decisions.^22^

### The Trial of Home Visits

The home visits cluster randomized controlled trial took place in Toro Local Government Area (LGA), the largest LGA in Bauchi State, with a projected population of about 490,000 in 18 wards (smallest administrative unit). Other papers document the trial methods and overall impacts on maternal and child health and male attitudes and actions.^17-20^ The study took place in eight wards of Toro LGA, chosen as being relatively secure and accessible. They have similar demographics and health metrics, and all have urban, rural, and rural-remote communities, in varying proportions. The mean number of households per ward was 5362 (range 2699 to 10,505). In a stepped-wedge design, households in six wards received home visits, in waves of two wards starting at 1-year intervals. We compared visited wards (intervention) with the baseline of the next wave wards (control). The two wards in the fourth wave did not go on to receive visits. Households in the pre-intervention (control) wards received normal government health services during their pre-intervention period.

In the intervention wards, women home visitors visited all households every 2 months. They identified and followed all pregnant women, sharing information from a recent local survey about risk factors for pregnancy and childbirth actionable at household level: lack of knowledge of danger signs during pregnancy and childbirth, continued heavy work in pregnancy, experience of violence during pregnancy, and lack of spousal communication about pregnancy and childbirth.^23^ In each two-monthly visit, the home visitor discussed with the pregnant woman what could be done in the household about the risk factors and asked them what had been done about them since the last visit. The home visitor shared with the woman information about danger signs during pregnancy and childbirth. Men home visitors separately visited the spouses of all the pregnant women, also every 2 months, and shared and discussed the same evidence in the same way. During the 12 months period in the intervention wards, the women home visitors collected information about each completed pregnancy and birth (including information about the risk factors) when they visited after the birth. At the start of the intervention in the next wave of wards, women home visitors collected baseline information about pregnancies and births during the preceding 12 months and about actionable risk factors.

Previously published analysis of maternal outcomes showed an overall impact of the intervention on morbidity during pregnancy and childbirth^18^ as well as an impact on the actionable risk factors of lack of knowledge of danger signs, continued heavy work in pregnancy, experience of violence during pregnancy, and lack of spousal communication about pregnancy and childbirth.^23^

### Efforts to Promote Equity

By design, the home visits were universal within the intervention wards. An initial mapping exercise, in collaboration with community leaders and the primary health care department of the LGA, identified all the households within the home visitor catchment areas, which covered all the communities within each intervention ward. The home visitors used GPS-enabled Android handsets to record information, allowing the research team to monitor their visits (location and timing) to all households. Home visitors received remuneration according to the records of visits they submitted electronically to the central server. In the early fieldwork, data managers, assisted by an algorithm, identified invalid records sent to the server and rejected them, requiring the visitor to submit a valid record for the visit to be eligible for payment. Demonstration to home visitors of the ability to track visits and identify invalid records reduced the frequency of invalid records from 16% to less than 1%. We later developed software housed on the handsets that prevented submission of invalid records to the server. To encourage visits to households in remote communities, we recruited and trained home visitors from intervention communities or nearby and paid an extra incentive for visits to households in remote communities.

### Equity Evaluation

The equity evaluation analysed *implementation* of the intervention, and *impact* of the intervention (on maternal outcomes and actionable risk factors), between levels of equity factors. We defined three equity factors at community, household, and individual level. For type of *community*, we compared urban with rural and rural-remote communities. Self-reported *household* food insecurity, defined as not having enough food in the last 2 weeks, served as an indicator of extreme poverty. For education of *individual* pregnant women, we compared those with and without any formal education. To assess equity of implementation of the home visits, we analysed data from visited pregnant women. We compared the proportion of pregnant women who had three or more visits during their pregnancy between levels of the three equity factors. We chose three visits as the benchmark for good implementation of the intervention because it reflected good follow-up of the pregnancy (the visits were intended to be two-monthly over the 9 months).

We used CIETmap open-source software,^24^ providing an interface with the R programming language, to calculate strength and statistical significance of associations as the odds ratio (OR) and 95% confidence interval (CI). The primary analysis of the association of each equity factor with the outcome of 3+ visits was bivariate, with supplementary multivariate analyses using the Mantel Haenszel procedure^25^ to control for the effects of age of the pregnant woman (adolescent or older) and education of the household head. To assess the pro-equity impact of the home visits, we compared maternal health outcomes and actionable risk factors between women in intervention and pre-intervention (control) wards, with stratification by the three equity factors to examine their modification of the intervention effect. In this analysis, we used the Lamothe cluster adjustment^26^ to allow for the allocation of the intervention by ward and the Zelen test for heterogeneity to test for significance of the effect modification.^27^

## Results

Table 1 shows the three equity factors in intervention and control wards. About 45% of the women (7212/15912) lived in urban communities; this proportion was slightly higher in intervention wards (48.4% vs 42.4%). Slightly fewer women in intervention wards lived in remote communities (11.8% vs 17.9%). The great majority of women (97%) lived in households with enough food in the last 2 weeks. Just over half (53%) of the women had some formal education, slightly more in intervention wards (54.7% vs 51.7%).

**Table 1.** Equity Factors Among Pregnant Women in Intervention and Pre-Intervention Wards.

Equity factor	% (fraction) of women		
	In intervention wards	In pre-intervention wards	All
Urban (vs rural, remote)	48.4 (3722/7684)	42.4 (3490/8228)	45.3 (7212/15912)
Remote (vs rural, urban)	11.8 (908/7684)	17.9 (1476/8228)	15.0 (2384/15912)
Household had enough food in last 2 weeks	96.9 (7297/7531)	97.3 (7735/7953)	97.1 (15032/15484)
Woman with any formal education	54.7 (4195/7676)	51.7 (4241/8211)	53.1 (8436/15887)

### Equity of Implementation of Home Visits

Among the 7684 pregnant women registered and visited at least once in intervention wards, 57.2% (4395) had three or more visits during their pregnancy. Table 2 shows the proportions of pregnant women with three or more visits by different levels of equity factors. Women in urban communities were 1.19 times more likely to have at least three visits compared with women in rural and rural-remote communities (OR 1.19, 95% CI 1.08-1.30). However, women in rural-remote communities were just as likely to have three or more visits compared with women in urban and rural communities (OR 1.00, 95% CI 0.87-1.15). Women from less poor households (enough food in the last 2 weeks) were 1.20 times more likely to have at least three visits compared with women from the poorest households (not enough food in the last 2 weeks), but the difference was not significant at the 5% level (OR 1.20, 95% CI 0.93-1.56). Women with some formal education were less likely to have at least three visits compared with women with no formal education (OR 0.89, 95% CI 0.81-0.98).

**Table 2.** Number of Visits to Pregnant Women for Different Levels of Equity Factors (Among 7684 Women in Intervention Wards Visited at Least once).

Equity factor		% (fraction) of women with 3+ visits	OR (95% CI)
Type of community (urban)	Urban	59.3 (2209/3722)	**1.19 (1.08-1.30)**
	Rural & rural-remote	55.2 (2186/3962)	
Type of community (remote)	Rural-remote	57.2 (519/908)	1.00 (0.87-1.15)
	Urban & rural	57.2 (3876/6776)	
Food security in last 2 weeks	Enough food	57.5 (4199/7297)	1.20 (0.93-1.56)
	Not enough food	53.0 (124/234)	
Education of woman	Some formal education	55.9 (2347/4195)	**0.89 (0.81-0.98)**
	No formal education	58.8 (2046/3481)	

OR = odds ratio, 95% CI = 95% confidence interval.

**Bold font** indicates an association significant at the 5% level.

Including the effects of age of the pregnant woman (adolescent vs older) and education of the household head in a supplementary multivariate analysis and analysing the effects of all three equity factors together did not materially change the findings shown in Table 2. Supplemental tables S1 and S2 show the results of the supplementary multivariate analyses.

### Pro-Equity Impact on Maternal Health Outcomes

Prior to the intervention, risk factors for maternal health (lack of knowledge of danger signs in pregnancy and childbirth, continued heavy work in third trimester, no discussion of pregnancy with spouse, physical violence in pregnancy) and maternal health outcomes (no persistent headache in pregnancy, no swelling of hands and feet in pregnancy and no post-natal sepsis) were generally worse among women in rural and rural-remote communities, among women from the poorest (food insecure) households, and among women without any formal education. Supplemental tables S3-S5 show details of the associations between the three equity factors and baseline risk factors for maternal health and maternal health outcomes in pre-intervention wards.

The analysis of the effect of equity factors on the impact of home visits included 7684 women visited at least once in the intervention wards and 8228 women in the pre-intervention wards. The impact of the home visits program on most risk factors for maternal health and maternal health outcomes was significantly greater among women living in rural and rural remote communities than among women living in urban communities (Table 3). The difference in impact by type of community was significant at the 5% level for knowledge of danger signs during pregnancy (OR 2.97 vs OR 4.63, Chi sq 43.44) and childbirth (OR 2.62 vs OR 6.42, Chi sq 84.43), reduction in heavy work before the third trimester (OR 1.56 vs OR 3.29, Chi sq 129.11), spousal communication about pregnancy and childbirth (OR 1.36 vs OR 2.81, Chi sq 107.54), swelling of hands and face during pregnancy (OR 2.10 vs OR 3.02, Chi sq 16.13), and post-natal sepsis (OR 1.41 vs OR 2.29, Chi sq 53.05). The impact of the visits on reduction of violence in pregnancy was slightly greater among women in urban communities (OR 1.96 vs OR 1.64, Chi sq 1.32) and the impact of the visits on persistent headache in pregnancy was slightly less in urban communities (OR 3.48 vs OR 3.93, Chi sq 2.97) but these differences were not significant at the 5% level.

**Table 3.** Impact of the Home Visits Intervention on Risk Factors for Maternal Health and Maternal Health Outcomes Among Women in Urban and Rural/Rural-Remote Communities.

Outcome	Women in urban communities			Women in rural and rural-remote communities			Test for heterogeneity
	% (fraction)			% (fraction)			
	Intervention	Control	OR (95%CIca)	Intervention	Control	OR (95%CIca)	Chi sq (*p* value)
Risk factors for maternal health							
Know 3+ pregnancy danger signs	62.1 (2313/3722)	35.6 (1242/3490)	2.97 (1.57-5.63)	59.7 (2364/3962)	24.2 (1147/4738)	4.63 (2.36-9.08)	**43.44 (0.00)**
Know 3 childbirth danger signs	26.8 (999/3722)	12.3 (428/3490)	2.62 (1.01-6.82)	24.77 (978/3962)	4.9 (230/4738)	6.42 (2.63-15.68)	**84.43 (0.00)**
Reduced work before 3rd trimester	49.3 (1808/3666)	38.5 (1289/3351)	1.56 (0.67-3.62)	56.4 (2186/3874)	28.3 (1298/4593)	3.29 (2.05-5.26)	**129.11 (0.00)**
Often discussed pregnancy with spouse	35.2 (1304/3701)	28.5 (979/3432)	1.36 (0.42-4.39)	41.1 (1619/3942)	19.9 (930/4674)	2.81 (1.50-5.24)	**107.54 (0.00)**
No physical violence in pregnancy	96.6 (3571/3697)	93.5 (3233/3456)	1.96 (1.11-3.44)	96.3 (3795/3942)	94.0 (4432/4713)	1.64 (0.88-3.06)	1.32 (0.25)
Maternal health outcomes							
No persistent headache in pregnancy	77.5 (2886/3722)	49.8 (1738/3490)	3.48 (1.22-9.93)	78.6 (3114/3962)	48.3 (2288/4738)	3.93 (1.94-7.97)	2.97 (0.09)
No swelling of hands or face in pregnancy	88.9 (3308/3722)	79.2 (2764/3490)	2.10 (1.43-3.07)	89.8 (3558/3962)	74.4 (3527/4738)	3.02 (1.64-5.58)	**16.13 (0.00)**
No post-natal sepsis	65.5 (2433/3717)	57.2 (1998/3490)	1.41 (0.79-2.53)	70.7 (2783/3934)	51.4 (2432/4736)	2.29 (1.56-3.39)	**53.05 (0.00)**

OR = odds ratio, 95%CIca = cluster adjusted 95% confidence interval, with ward as the cluster.

**Bold font** indicates a difference in OR between the strata (urban and rural/rural-remote communities) significant at the 5% level.

Using household food insecurity as an indicator of serious poverty, the home visits had a greater impact on risk factors for maternal health and maternal health outcomes (except swelling of hands and face in pregnancy) among women from the poorest households than among women from less poor households (Table 4). This difference in impact was significant at the 5% level for knowledge about danger signs during pregnancy (OR 3.69 vs OR 8.52, Chi sq 15.01) and childbirth (OR 3.78 vs OR 13.00, Chi sq 13.20), reduction in heavy work before the third trimester (OR 2.29 vs OR 3.69, Chi sq 5.33), and no persistent headache (as a sign of pre-eclampsia) (OR 3.70 vs OR 5.64, Chi sq 3.70).

**Table 4.** Impact of the Home Visits Intervention on Risk Factors for Maternal Health and Maternal Health Outcomes Among Women in Households With and Without Food Security.

Outcome	Women in households with enough food in last 2 weeks			Women in households without enough food in last 2 weeks			Test for heterogeneity
	% (fraction)			% (fraction)			
	Intervention	Control	OR (95%CIca)	Intervention	Control	OR (95%CIca)	Chi sq (*p* value)
Risk factors for maternal health							
Know 3+ pregnancy danger signs	60.4 (4409/7297)	29.3 (2263/7735)	3.69 (2.16-6.29)	70.1 (164/234)	21.6 (47/218)	8.52 (3.58-20.31)	**15.01 (0.00)**
Know 3 childbirth danger signs	25.2 (1840/7297)	8.2 (634/7735)	3.78 (1.62-8.79)	38.5 (90/234)	4.6 (10/218)	13.00 (2.20-76.86)	**13.20 (0.00)**
Reduced work before 3rd trimester	52.6 (3764/7160)	32.6 (2435/7465)	2.29 (1.31-4.01)	55.7 (128/230)	25.4 (54/213)	3.69 (1.21-11.27)	**5.33 (0.02)**
Often discussed pregnancy with spouse	37.9 (2752/7259)	23.5 (1792/7623)	1.99 (1.03-3.83)	44.6 (103/231)	23.4 (50/214)	2.64 (1.22-5.72)	1.80 (0.18)
No physical violence in pregnancy	96.6 (7007/7256)	94.1 (7226/7680)	1.77 (1.17-2.66)	93.5 (217/232)	87.5 (189/216)	2.07 (0.73-5.86)	0.20 (0.65)
Maternal health outcomes							
No persistent headache in pregnancy	77.9 (5684/7297)	48.8 (3771/7735)	3.70 (1.75-7.85)	81.6 (191/234)	44.0 (96/218)	5.64 (2.35-13.57)	**3.70 (0.054)**
No swelling of hands or face in pregnancy	89.4 (6524/7297)	76.1 (5883/7735)	2.66 (1.37-5.14)	89.3 (209/234)	79.4 (173/218)	2.17 (0.99-4.80)	0.54 (0.46)
No post-natal sepsis	68.1 (4947/7265)	53.8 (4157/7733)	1.84 (1.14-2.96)	67.8 (158/233)	50.5 (110/218)	2.07 (1.23-3.48)	0.36 (0.55)

OR = odds ratio, 95%CIca = cluster adjusted 95% confidence interval, with ward as the cluster.

**Bold font** indicates a difference in OR between the strata (household with and without enough food in the last 2 weeks) significant at the 5% level.

Table 5 shows the impact of the home visits on risk factors for maternal health and maternal health outcomes among women with and without any formal education. The impact was significantly greater among women with no formal education for knowledge of danger signs during pregnancy (OR 3.33 vs OR 4.63, Chi sq 29.03) and childbirth (OR 3.09 vs OR 5.28, Chi sq 30.88), spousal communication about pregnancy (OR 1.82 vs OR 2.32, Chi sq 10.94), and swelling of hands and face during pregnancy (OR 2.22 vs OR 3.05, Chi sq 12.03). For reduction of violence during pregnancy, the impact of the program was significantly greater among women who had some formal education (OR 2.11 vs OR 1.48, Chi sq 5.41).

**Table 5.** Impact of the Home Visits Intervention on Risk Factors for Maternal Health and Maternal Health Outcomes Among Women With and Without Any Formal Education.

Outcome	Women with any formal education			Women without any formal education			Test for heterogeneity
	% (fraction)			% (fraction)			
	Intervention	Control	OR (95%CIca)	Intervention	Control	OR (95%CIca)	Chi sq (*p* value)
Risk factors for maternal health							
Know 3+ pregnancy danger signs	58.2 (2441/4915)	30.2 (1281/4241)	3.22 (1.82-5.67)	64.1 (2232/3481)	27.9 (1106/3970)	4.63 (2.66-8.06)	**29.03 (0.00)**
Know 3 childbirth danger signs	22.4 (941/4195)	8.6 (363/4241)	3.09 (1.35-7.06)	29.7 (1033/3481)	7.4 (294/3970)	5.28 (1.98-14.06)	**30.88 (0.00)**
Reduced work before 3rd trimester	55.9 (2312/4134)	36.4 (1494/4108)	2.22 (1.15-4.30)	49.4 (1679/3398)	28.4 (1084/3820)	2.47 (1.37-4.42)	2.44 (0.12)
Often discussed pregnancy with spouse	44.4 (1851/4166)	30.5 (1274/4175)	1.82 (1.04-3.18)	30.9 (1071/3471)	16.1 (632/3914)	2.32 (1.02-5.27)	**10.94 (0.00)**
No physical violence in pregnancy	96.8 (4031/4164)	93.5 (3950/4225)	2.11 (1.26-3.55)	96.0 (3328/3467)	94.2 (3699/3927)	1.48 (0.89-2.44)	**5.41 (0.02**)
Maternal health outcomes							
No persistent headache in pregnancy	78.8 (3305/4195)	50.3 (2135/4241)	3.66 (1.71-7.84)	77.3 (2690/3481)	47.4 (1883/3970)	3.77 (1.73-8.20)	0.16 (0.69)
No swelling of hands or face in pregnancy	88.9 (3730/4195)	78.3 (3321/4241)	2.22 (1.07-4.62)	89.9 (3129/3481)	74.5 (2957/3970)	3.05 (1.60-5.78)	**12.03 (0.00)**
No post-natal sepsis	70.9 (2964/4180)	50.3 (1995/3968)	1.83 (1.21-2.77)	64.8 (2246/3464)	50.3 (1995/3968)	1.82 (0.97-3.43)	0.00 (0.98)

OR = odds ratio, 95%CIca = cluster adjusted 95% confidence interval, with ward as the cluster.

**Bold font** indicates a difference in OR between the strata (with and without formal education) significant at the 5% level. For the outcome of “No physical violence in pregnancy” the OR was significantly higher among women *with* formal education.

## Discussion

Implementation of the home visits intervention was equitable across the three domains of social disadvantage we studied. Women in rural-remote communities and those in extremely poor households (not enough food in last 2 weeks) were no less likely to receive three visits than those in in less remote and less poor situations, and women without formal education were slightly more likely to receive three visits than women with formal education. The home visits had a pro-equity impact, with a stronger effect on actionable risk factors for maternal health and on a range of maternal health outcomes among disadvantaged women.

In their systematic review of pro-equity impacts of interventions for maternal and child health in LMICs, Yuan et al^14^ called for more description of the implementation of the interventions. In this report, we describe the specific efforts to promote equity of implementation of the home visits program. The truly *universal* nature of the program was crucial, emphasizing efforts to reach every last pregnant woman. Without such an emphasis, disadvantaged groups might well not be reached by a health program even when it includes outreach. A systematic review of community interventions to improve birth preparedness and complications readiness in LMICs concluded that interventions reaching more than 30% of the target population were more effective.^28^ We argue that programs must reach very much more than 30% to promote equity, as the last 10% to be reached by non-universal services suffer much more than 10% of the health burdens. The inverse care law expounded by Julian Tudor Hart^29^ over 50 years ago is still highly relevant in LMICs today:^30^ those most in need of health services are least likely to have access to them.

Ensuring that health interventions reach all who need them is a daunting task. An important technical aid to full coverage of the Toro home visits was remote monitoring via the GPS-enabled handsets of the visitors. Digital monitoring of health and other workers is increasing and has the potential to be misused as covert and intrusive surveillance with invasion of privacy.^31^ In our pro-equity application, digital monitoring replaced human supervisors who, while adding another layer that itself requires monitoring, can never check more than a small proportion of reported visited households. We initially identified invalid records after they were sent to the central server. This required considerable personnel time to check each flagged invalid record; our subsequent software fix prevented submission of invalid records, making remote monitoring and quality control feasible for home visits as part of a routine service to reach all households. The Bauchi State government has committed to such a service when funding is available and released.

The home visits intervention had greater impact on several outcomes among more disadvantaged women: those living in rural areas, from the poorest households, and without formal education. These disadvantaged women had worse outcomes than other women prior to the intervention. Other authors have reported on the pro-equity impact of community interventions. A meta-analysis of four cluster randomised controlled trials of participatory community women’s groups with more than 30% coverage of the target populations in South Asia and Africa found the impact on neonatal mortality was stronger in the most marginalized populations.^32^ Focus group discussions linked to these trials suggested that the women’s groups increased knowledge and confidence to act on issues that could be addressed within the household; this allowed even the most marginalized households to improve their situation.^33^ Two systematic reviews concluded that community interventions could have a pro-equity effect on service use and some child health outcomes, especially those with high coverage, with a focus on actions households could take themselves, and provision of services within communities.^15,16^ In the Toro intervention, home visitors shared information about local maternal health risk factors that were actionable at household level and they asked about these actions in the two-monthly repeat visits throughout the pregnancy. The visits overall had a strong impact on these identified upstream risk factors,^18^ and our equity analysis shows this impact was stronger among the more disadvantaged women. We do not suggest that home visits stimulating household actions are a replacement for improving access to high quality antenatal and institutional childbirth, but they can benefit women, especially disadvantaged women, in a context of limited access to quality maternal care services. A greater benefit could accrue if services were also improved.

We believe the home visits had an equity impact for three reasons. First, the *true* universal coverage of communities and households, with effective remote monitoring to ensure this happened in practice, meant the intervention really did reach the most marginalized women. Second, the visits shared evidence about local risk factors that were *actionable by households*, rather than simply encouraging women to attend for routine antenatal care and institutional childbirth with all the inequities of access that entails,^9,34,35^ as well as the often inadequate quality of the care available.^36,37^ Stories of change from women and men in intervention households illustrated how the home visits made them feel empowered to act themselves to tackle the risk factors, whatever their educational or socio-economic status.^38^ Reported actions in households included making time for discussions between spouses, providing help so that women did not need to continue heavy work in pregnancy, learning how to avoid violence in response to disagreements, and planning actions in the event of danger signs during pregnancy and childbirth. Third, in addition to women home visitors interacting with the pregnant women, men home visitors interacted with their spouses. They shared the same local evidence about risk factors, all of them actionable by men, and discussed with them on repeat visits the actions they were taking. This stimulated household-level discussions after the home visitors had left. It strengthened household actions not dependent on health services in a context of men being responsible for most household decisions. Narratives of change suggested that the home visits intervention promoted gender equity and in some cases it was gender transformative.^39^

### Strengths and Limitations

Available data about numbers of visits during the pregnancy allowed us to compare the intensity of the intervention between levels of equity factors as a measure of equity of implementation beyond simple coverage. The strong study design of the randomized controlled trial allowed us to examine the effect modification of impact by different equity factors with less concern about confounding by other variables. Our equity analysis is limited by the single question about household food sufficiency as an indicator of extreme poverty, rather than a set of questions to create a scale of economic status. However, we have found the response to this question is a good indicator of serious poverty in the context of Bauchi and other resource-poor settings.^40,41^ Maternal health outcomes were self-reported, but we have no reason to believe they would be reported differently according to the level of the equity factors we examined.

## Conclusion

Universal home visits to pregnant women and their spouses had equitable implementation and a pro-equity impact on risk factors for maternal health and maternal health outcomes. The universal coverage, supported by remote monitoring, and discussion of local risk factors actionable at household level with both pregnant women and their male spouses, contributed to the equity impact. Truly universal household level programs can help overcome barriers to accessing services from health facilities and are a feasible approach to support universal health coverage.

## Supplemental Material


Supplemental Material - The Equity Impact of Universal Home Visits to Pregnant Women and Their Spouses in Bauchi State, Nigeria: Secondary Analysis From a Cluster Randomised Controlled Trial
Supplemental Material for The Equity Impact of Universal Home Visits to Pregnant Women and Their Spouses in Bauchi State, Nigeria: Secondary Analysis From a Cluster Randomised Controlled Trial by Anne Cockcroft, Loubna Belaid, Khalid Omer, Umaira Ansari, Amar Aziz, Yagana Gidado, Hadiza Mudi, Rilwanu Mohammed, Rakiya Sale and Neil Andersson in Community Health Equity Research & Policy.
