# Supplementary material for: The Equity Impact of Universal Home Visits to Pregnant Women and Their Spouses in Bauchi State, Nigeria: Secondary Analysis From a Cluster Randomised Controlled Trial
Source: Community Health Equity Res Policy. 2024 Apr 27;45(2):141–51. doi: 10.1177/2752535X241249893 (PMC11577682; doi:10.1177/2752535X241249893)
Supplement: Supplemental Material - The Equity Impact of Universal Home Visits to Pregnant Women and Their Spouses in Bauchi State, Nigeria: Secondary Analysis From a Cluster Randomised Controlled Trial [file sj-pdf-1-qch-10.1177_2752535X241249893.pdf]

Supplementary Table S1. Effects of equity factors on the outcome of pregnant women having 3+ visits (among 7684 women in intervention wards) adjusting for effects of other variables

(a) Urban vs rural and rural-remote communities

Variable	Crude OR	Adjusted OR	95% CI adjusted OR
Urban vs rural & rural-remote community	1.20	1.21	**1.11 - 1.33**
Adolescent vs older pregnant woman	0.91	0.88	0.76 - 1.01
Educated vs uneducated household head	0.99	0.97	0.88 - 1.06

(b) Rural-remote vs urban and rural communities

Variable	Crude OR	Adjusted OR	95% CI adjusted OR
Rural-remote vs urban & rural communities	1.05	1.04	0.90 - 1.20
Adolescent vs older pregnant woman	0.91	0.91	0.79 - 1.05
Educated vs uneducated household head	0.99	0.99	0.90 - 1.09

(c) Household food security in last two weeks (as reflection of poverty)

Variable	Crude OR	Adjusted OR	95% CI adjusted OR
Enough vs not enough food in last 2 weeks	1.19	1.18	0.91 - 1.54
Adolescent vs older pregnant woman	0.90	0.91	0.79 - 1.04
Educated vs uneducated household head	0.99	0.98	0.90 - 1.08

(d) Education of pregnant woman

Variable	Crude OR	Adjusted OR	95% CI adjusted OR
Some vs no education of pregnant woman	0.89	0.88	**0.79 - 0.97**
Adolescent vs older pregnant woman	0.91	0.90	0.78 - 1.03
Educated vs uneducated household head	0.98	1.03	0.93 - 1.14

Crude OR=odds ratio for the association of the variable with the outcome (3+ home visits) without adjustment for effects of the other variables
Adjusted OR=odds ratio for the association of the variable with the outcome (3+ home visits) adjusted for effects of the other variables
95% CI adjusted OR=95% confidence interval around the adjusted odds ratio

Bold font indicates an association between the variable and the outcome (3+ home visits) adjusted for effects of the other variables significant at the 5% level.

Supplementary Table S2. Simultaneous effects of three equity factors on the outcome of pregnant women having 3+ visits (among 7684 women in intervention wards)

Equity factor	Crude OR	Adjusted OR	95% CI adjusted OR
Urban vs rural & rural-remote community	1.18	1.20	**1.10 - 1.32**
Enough vs not enough food in last 2 weeks	1.20	1.18	0.91 - 1.53
Some vs no education of pregnant woman	0.89	0.86	**0.78 - 0.94**

Crude OR=odds ratio for the association of the variable with the outcome (3+ home visits) without adjustment for effects of the other variables
Adjusted OR=odds ratio for the association of the variable with the outcome (3+ home visits) adjusted for effects of the other variables
95% CI adjusted OR=95% confidence interval around the adjusted odds ratio

Bold font indicates an association between the variable and the outcome (3+ home visits) adjusted for effects of the other variables significant at the 5% level.

Supplementary Table S3. Baseline risk factors for maternal health and maternal health outcomes among women living in urban and rural/rural-remote communities (among 8228 women in pre-intervention wards)

Outcome	% (fraction) of women		
	In urban communities	In rural/rural-remote communities	OR (95% CI)
Risk factors for maternal health			
Know 3+ pregnancy danger signs	35.6 (1242/3490)	24.2 (1147/4738)	**1.73 (1.57-1.90)**
Know 3 childbirth danger signs	12.3 (428/3490)	4.9 (230/4738)	**2.74 (2.33-3.22)**
Reduced work before 3rd trimester	38.5 (1289/3351)	28.3 (1298/4593)	**1.59 (1.44-1.74)**
Often discussed pregnancy with spouse	28.5 (979/3423)	19.9 (930/4674)	**1.61 (1.45-1.78)**
No physical violence in pregnancy	93.5 (3233/3456)	94.0 (4432/4713)	0.92 (0.77-1.10)
Maternal health outcomes			
No persistent headache in pregnancy	49.8 (1738/3490)	48.3 (2288/4738)	1.06 (0.97-1.16)
No swelling of hands or face in pregnancy	79.2 (2764/3490)	74.4 (3527/4738)	**1.31 (1.18-1.45)**
No post-natal sepsis	57.2 (1998/3490)	51.4 (2432/4736)	**1.27 (1.16-1.39)**

OR=odds ratio, 95% CI=95% confidence interval.

The odds ratio relates to the association between type of community (urban vs rural and rural-remote) and absence of the risk factor. For example, women in urban communities were 1.73 times more likely to know three or more danger signs during pregnancy compared with women in rural and rural-remote communities.

Bold font indicates an association between the risk factor and type of community significant at the 5% level.

Supplementary Table S4. Baseline risk factors for maternal health and maternal health outcomes among women living in households with and without enough food in the last two weeks (among 8228 women in pre-intervention wards)

Outcome	% (fraction) of women in households		
	With enough food in last 2 weeks	Without enough food in last 2 weeks	OR (95% CI)
Risk factors for maternal health			
Know 3+ pregnancy danger signs	29.3 (2263/7735)	21.6 (47/218)	**1.50 (1.09-2.08)**
Know 3 childbirth danger signs	8.2 (634/7735)	4.6 (10/218)	**1.86 (0.99-3.49)**
Reduced work before 3rd trimester	32.6 (2435/7465)	25.4 (54/213)	**1.43 (1.04-1.95)**
Often discussed pregnancy with spouse	23.5 (1792/7623)	23.4 (50/214)	1.01 (0.73-1.39)
No physical violence in pregnancy	94.1 (7226/7680)	87.5 (189/216)	**2.27 (1.52-3.40)**
Maternal health outcomes			
No persistent headache in pregnancy	48.8 (3771/7735)	44.0 (96/218)	1.21 (0.92-1.58)
No swelling of hands or face in pregnancy	76.1 (5883/7735)	79.4 (173/218)	0.83 (0.56-1.14)
No post-natal sepsis	53.8 (4157/7733)	50.5 (110/218)	1.14 (0.87-1.49)

OR=odds ratio, 95% CI=95% confidence interval.

The odds ratio relates to the association between household food security (as an indicator of poverty) and absence of the risk factor. For example, women in food secure households were 1.50 times more likely to know three or more danger signs during pregnancy compared with women in food insecure households.

Bold font indicates an association between the risk factor and household food security significant at the 5% level.

Supplementary Table S5. Baseline risk factors for maternal health and maternal health outcomes among women with and without any formal education (among 8228 women in pre-intervention wards)

Outcome	% (fraction) of women		
	With any formal education	Without any formal education	OR (95% CI)
Risk factors for maternal health			
Know 3+ pregnancy danger signs	30.2 (1281/4241)	27.9 (1106/3970)	**1.12 (1.02-1.23)**
Know 3 childbirth danger signs	8.6 (363/4241)	7.4 (294/3970)	**1.17 (1.00-1.37)**
Reduced work before 3rd trimester	36.4 (1494/4108)	28.4 (1084/3820)	**1.44 (1.31-1.59)**
Often discussed pregnancy with spouse	30.5 (1274/4175)	16.1 (632/3914)	**2.28 (2.05-2.54)**
No physical violence in pregnancy	93.5 (3950/4225)	94.2 (3699/3927)	0.89 (0.74-1.06)
Maternal health outcomes			
No persistent headache in pregnancy	50.3 (2135/4241)	47.4 (1883/3970)	**1.12 (1.03-1.23)**
No swelling of hands or face in pregnancy	78.3 (3321/4241)	74.5 (2957/3970)	**1.24 (1.12-1.37)**
No post-natal sepsis	57.2 (2424/4241)	50.3 (1995/3968)	**1.32 (1.21-1.44)**

OR=odds ratio, 95% CI=95% confidence interval.

The odds ratio relates to the association between education of the pregnant women (any vs no formal education) and absence of the risk factor. For example, women with any formal education were 2.28 times more likely to have often discussed pregnancy and childbirth with their spouse compared with women without any formal education.

Bold font indicates an association between the risk factor and education of the pregnant woman significant at the 5% level.
